# Mapping of a Regulatory Site of the *Escherichia coli* ADP-Glucose Pyrophosphorylase

**DOI:** 10.3389/fmolb.2019.00089

**Published:** 2019-09-25

**Authors:** Jaina A. Bhayani, Benjamin L. Hill, Anisha Sharma, Alberto A. Iglesias, Kenneth W. Olsen, Miguel A. Ballicora

**Affiliations:** ^1^Department of Chemistry and Biochemistry, Loyola University Chicago, Chicago, IL, United States; ^2^Laboratorio de Enzimología Molecular, Instituto de Agrobiotecnología del Litoral (UNL-CONICET), CCT CONICET, Santa Fe, Argentina

**Keywords:** allosteric regulation, polysaccharide biosynthesis, pyridoxal 5′-phosphate activation, sugar phosphate regulation, arginine scanning mutagenesis

## Abstract

The enzyme ADP-glucose pyrophosphorylase (ADP-Glc PPase) controls the biosynthesis of glycogen in bacteria and starch in plants. It is regulated by various activators in different organisms according to their metabolic characteristics. In *Escherichia coli*, the major allosteric activator is fructose 1,6-bisphosphate (FBP). Other potent activator analogs include 1,6-hexanediol bisphosphate (HBP) and pyridoxal 5**′**-phosphate (PLP). Recently, a crystal structure with FBP bound was reported (PDB ID: 5L6S). However, it is possible that the FBP site found is not directly responsible for the activation of the enzyme. We hypothesized FBP activates by binding one of its phosphate groups to another site (“P1”) in which a sulfate molecule was observed. In the *E. coli* enzyme, Arg40, Arg52, and Arg386 are part of this “P1” pocket and tightly complex this sulfate, which is also present in the crystal structures of ADP-Glc PPases from *Agrobacterium tumefaciens* and *Solanum tuberosum*. To test this hypothesis, we modeled alternative binding conformations of FBP, HBP, and PLP into “P1.” In addition, we performed a scanning mutagenesis of Arg residues near potential phosphate binding sites (“P1,” “P2,” “P3”). We found that Arg40 and Arg52 are essential for FBP and PLP binding and activation. In addition, mutation of Arg386 to Ala decreased the apparent affinity for the activators more than 35-fold. We propose that the activator binds at this “P1” pocket, as well as “P2.” Arg40 and Arg52 are highly conserved residues and they may be a common feature to complex the phosphate moiety of different sugar phosphate activators in the ADP-Glc PPase family.

## Introduction

**Figure 1:**
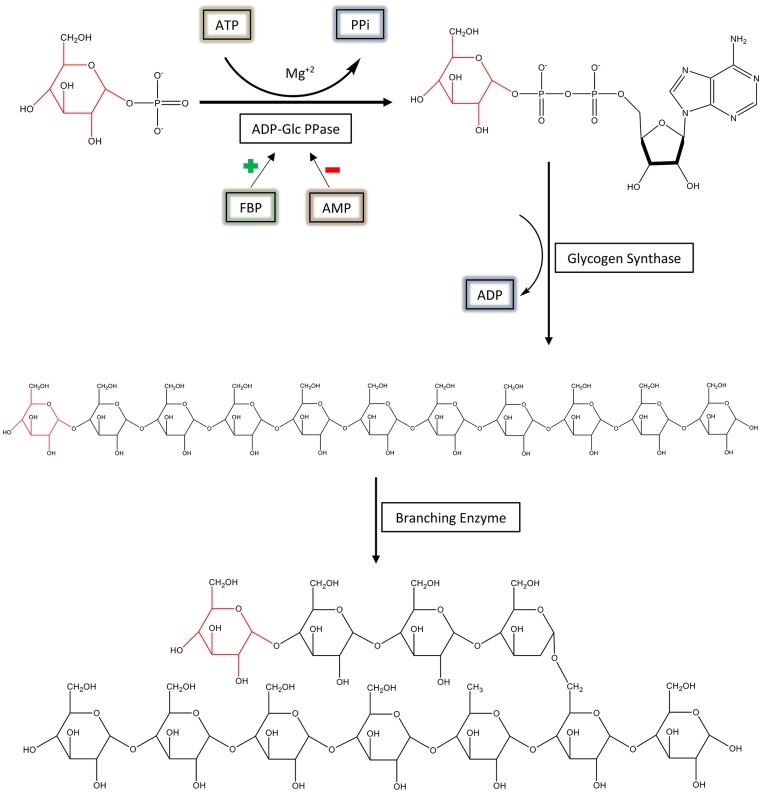
The regulation and synthesis of glycogen in *E. coli*. ADP-Glc PPase regulates the production of glycogen in bacteria. ADP-Glc PPase catalyzes the reaction between Glc1P and ATP in the presence of Mg^2+^, forming ADP-Glc and PPi. This reaction takes place under physiological conditions; however, the reaction is reversible *in vitro*. ADP-Glc PPase is activated by FBP and inhibited by AMP. The production of ADP-Glc leads to the synthesis of a linear α-1,4 glucan molecule by glycogen synthase, which is then branched with an α-1,6 linkage by the branching enzyme.

ADP-glucose pyrophosphorylase (EC 2.7.7.27; ADP-Glc PPase) is a regulatory, homotetrameric enzyme that catalyzes the reaction between adenosine triphosphate (ATP) and glucose 1-phosphate (Glc1P), producing ADP-glucose (ADP-Glc) and pyrophosphate (PPi) (Figure 1) (Ballicora et al., 2003). By synthesizing ADP-Glc, this enzyme plays a critical role in the production of glycogen and starch, important energy storage molecules in bacteria and plants, respectively (Ballicora et al., 2003). ADP-Glc PPase is allosterically regulated by metabolites that indicate the energy status of the cell. ADP-Glc PPases have been classified into several different groups based on their specificity toward different allosteric regulators (Ballicora et al., 2003, 2004). Enteric bacteria, such as *Escherichia coli*, are mainly activated by fructose 1,6-bisphosphate (FBP), the addition of which causes dramatic changes in the enzyme's kinetic profile (Hill et al., 2015). FBP affected both the *V*_max_ and affinity of substrates. FBP increases the enzyme's specific activity more than 20-fold with an additional decrease of the *S*_0.5_ for ATP (Ballicora et al., 2003, 2004; Hill et al., 2015). On the other hand, AMP competes with FBP, abolishing the activation effects of FBP (Gentner and Preiss, 1968). Traditionally, *E. coli* ADP-Glc PPase was categorized as an enzyme solely activated by FBP (Ballicora et al., 2003), but it was recently found that pyruvate is a secondary activator that synergistically enhances the effect of FBP (Asencion Diez et al., 2014). In absence of FBP, pyruvate only activates 3.3-fold at 50 mM (Asencion Diez et al., 2014). Because ADP-Glc PPase plays such a vital role in glycogen synthesis, it is necessary to develop a complete mechanical understanding of the enzyme's regulation. In addition, based on the strong effects of FBP, *E. coli* ADP-Glc PPase is an excellent case study for allosteric regulation (Hill et al., 2015).

The N-terminal region of diverse ADP-Glc PPases is involved in the regulation of enzymatic activity (Gardiol and Preiss, 1990; Preiss, 1996; Gomez-Casati et al., 2001). Sequence alignment reveals the presence of conserved arginine residues in the N-terminal region of similarly regulated ADP-Glc PPases. For example, Arg33 and Arg45 from *Agrobacterium tumefaciens* ADP-Glc PPase, are pivotal to the activation of the enzyme by fructose 6-phosphate (Fru6P) (Gomez-Casati et al., 2001). Furthermore, in the *E. coli* enzyme, Lys39 was described as an important residue for the activation by FBP (Gardiol and Preiss, 1990). In addition, Lys39 forms a Schiff base with pyridoxal 5′-phosphate (PLP), which works as an analog of FBP (Parsons and Preiss, 1978a,b). Mutagenesis in the *A. tumefaciens* enzyme indicated that the pyruvate regulatory site does not completely overlap with the Fru6P site (Gomez-Casati et al., 2001). This was in agreement with the structures of the enzymes from *E. coli* and *A. tumefaciens* with activators bound (FBP and pyruvate, respectively) (Cifuente et al., 2016; Hill et al., 2019).

Recently, the crystal structure of ADP-Glc PPase from *E. coli* was solved in the presence of the β-D-furanose form of FBP (PDB ID: 5L6S) (Cifuente et al., 2016). In this structure, the phosphates from FBP are interacting with Lys39, Arg130, and residues from the C-terminal domain (Arg419, Arg423, and backbone interactions with Gln429 and Glu430). However, prior studies indicated that the truncation of two C-terminal residues (Glu430 and Arg431) did not significantly affect the activation of the enzyme (Wu and Preiss, 1998, 2001). In addition, it was previously suggested that the activating conformation of FBP is the linear form rather than the furanose (Haugen and Preiss, 1979). We hypothesize that the FBP bound in the published structure (Cifuente et al., 2016) is at an alternative site, one that does not directly activate the enzyme. A possible explanation for this scenario is that the presence of sulfate in the crystallization conditions may have precluded the native binding of FBP by competing for the same site. Interestingly, in the *A. tumefaciens* ADP-Glc PPase, sulfate competes with another sugar phosphate activator, fructose 6-phosphate (Fru6P) (Cupp-Vickery et al., 2008). To test our hypothesis, we assayed whether sulfate competes with FBP activation. In addition, we modeled the structure of 1,6-hexanediol bisphosphate (HBP), which is a simple analog of linear FBP into the structure of the enzyme (Haugen and Preiss, 1979). HBP and FBP are analogs because not only do they activate the enzyme, but also FBP competes with the binding of 1,6-[^14^C_1_]hexanediol bisphosphate (Haugen and Preiss, 1979). In addition, both competed with the binding of radiolabeled AMP (Haugen and Preiss, 1979). Modeling of HBP was performed using the position of sulfates and other phosphate groups bound to the enzyme as guidelines (Cifuente et al., 2016). Afterwards, we mutated the Arg residues that were predicted to interact with HBP. Structural, binding, and kinetic analysis allowed us to conclude there is a site, occupied by a sulfate in the crystal structure, which is critical for activation of the enzyme.

## Materials and Methods

### Materials

Biochemicals used for activity assays were from Sigma-Aldrich (St. Louis, MO). *E. coli* BL21 (DE3) cells were purchased from New England BioLabs (Ipswich, MA). Bacterial growth media and antibiotics were provided by IBI Scientific (Pittsburgh, PA). All the other chemicals were of the highest quality available.

### Site-Directed Mutagenesis

The *E. coli* ADP-Glc PPase gene (*glgC*) was subcloned into pET28c (Novagen) to add an N-terminal hexahistidine-tagged protein. The coding fragment was obtained from pETEC (Ballicora et al., 2002) by digesting it with NdeI and SacI restriction enzymes and ligating it to a previously digested pET28c. The final construct (pETEC2) was used as a template for mutagenesis by using a Q5 Site-Directed Mutagenesis Kit from New England BioLabs. Oligonucleotides were synthesized by Integrated DNA Technologies (IDT, San Diego). The primers listed were used to make the following mutants: R40A forward 5′- AACCAATAAGgcaGCAAAACCGGCC-3′; R40A reverse 5′- AAATCCTTCAGGCGGGTA-3′; R52A forward 5′- CGGTAAGTTCgccATTATCGACTTTGCGCTGTC-3′; R52A reverse 5′-CCGAAGTGTACGGCCGGT-3′; R52K forward 5′- CGGTAAGTTCaagATTATCGACTTTGCGCTGTCTAAC-3′; R52K reverse 5′- CCGAAGTGTACGGCCGGT-3′; R130A forward 5′- CATTATCCGCgctTATAAAGCGGAATACGTG-3′; R130A reverse 5′-TCGAGGTTTTGGGTGACC-3′; R353A forward 5′- TCTGTTCTCGgccGTTCGCGTGAATTCATTCTG-3′; R353A reverse 5′- ACGGACTGCACCACCACC-3′; R386A forward 5′- CGTCATCGATgctGCTTGTGTTATTCCGGAAGGCATGG-3′; R386A reverse 5′- CAGCGGCGCAGACGGCAC-3′; R419A forward 5′- GCTGGTAACGgccGAAATGCTAC-3′; R419A reverse 5′- ACGATGCCTTCTTCTGAAC-3′; R423A forward 5′- CGAAATGCTAgccAAGTTAGGGCATAAACAGGAG-3′; R423A reverse: 5′- CGCGTTACCAGCACGATG-3′. All mutations were confirmed by automated DNA sequencing performed by the University of Chicago Cancer Research Center (CRC, Chicago).

### *E. coli* ADP-Glc PPase Expression and Purification

Transformed *E. coli* (BL21) cells, with mutant or wild type pETEC2, were grown in 2.8-liter Fernbach flasks containing 1 liter of Luria-Bertani Miller supplemented with 50 μg/ml of kanamycin. This was performed at 37°C and 250 rpm until the optical density at 600 nm (OD_600_) reached ~0.6. Protein expression was induced by the addition of 0.5 mM isopropyl-β-D-1-thiogalactopyranoside. Cells were incubated at 25°C and harvested after 16 h by centrifugation at 8,000 rpm and 4°C for 10 min. The cell pellet was re-suspended in buffer C (50 mM HEPES pH 8.0, 200 mM NaCl, 10% [vol/vol] glycerol, 10 mM imidazole) and disrupted by sonication. The resulting suspension was centrifuged twice at 20,000 rpm and 4°C for 15 min, and the soluble fraction was loaded onto a 5 ml HisTrap column (GE Life Sciences, Piscataway, NJ) containing Ni^2+^ and previously equilibrated with buffer C. Elution of the retained proteins was achieved with a linear imidazole gradient (30 column volumes, 10–325 mM). Wild type and mutant proteins were purified to apparent homogeneity as verified via SDS-PAGE. Fractions containing ADP-Glc PPase were pooled, concentrated to ~10 mg/ml, supplemented with 5% (vol/vol) glycerol, and stored at −80°C until use.

### Enzyme Assays

Activity of ADP-Glc PPase was measured in the direction of ADP-glucose synthesis by detecting phosphate in the colorimetric assay as previously described (Figueroa et al., 2011). The phosphate is generated after hydrolysis of the product pyrophosphate (PPi) with pyrophosphatase as indicated (Fusari et al., 2006). Reactions were performed in polystyrene flat-bottom microplates and the absorbance was measured at 620 nm in a Multiskan Ascent reader. Unless otherwise stated, assay conditions were as follows: an appropriate enzyme dilution was added to a mixture containing 50 mM HEPES pH 8.0, 10.0 mM MgCl_2_, 1.0 mM Glc1P, 1.5 mM ATP, 1.5 U/ml inorganic pyrophosphatase, 0.2 mg/ml bovine serum albumin, in a total volume of 25 μl. For assays varying inhibitor AMP concentrations, 0.18 mM FBP was present in assay mixtures. The unit of enzyme activity (U) is defined as 1.0 μmol of PPi formed per minute.

### Kinetic Parameters

Specific enzyme activity (U/mg) vs. effector concentration was plotted and fit to the following modified Hill equation: $V=v_{o}+\left(V_{\max}-v_{o}\right)\;X^{n_{H}}/\left(X_{0.5}^{n_{H}}+X^{n_{H}}\right)$ (Figueroa et al., 2011; Hill et al., 2019). The velocity in the absence of the effector being analyzed is *v*_0_, the velocity at saturating concentrations of the activator is *V*_max_, *X* is the concentration of the substrate ([S]) or activator ([A]) under study, *X*_0.5_ is the concentration of the substrate (*S*_0.5_) or activator (*A*_0.5_) needed to reach half of the maximum velocity, and *n*_H_ is the Hill coefficient. In the text, we refer to the reciprocal of the *S*_0.5_ and *A*_0.5_ as the apparent affinities for the ligands. Fitting was performed using the program Origin™ 8.0 with the Levenberg-Marquardt non-linear least-squares algorithm. The errors were obtained with that algorithm as well. All kinetic assays were performed at least in duplicate with reproducibility of parameters within ±10%.

### Thermal Shift Assays

Thermal shift assays were performed as described (Hill et al., 2019) using the Step One Real-Time PCR System^TM^ (Thermo Fisher Scientific) and Step One^TM^ software. The final volume for the assay was 20 μl and contained 50 mM HEPES (pH 8.0), SYPRO Orange Dye (Sigma-Aldrich) (4X) and 0.02 mg/ml purified protein, in the absence and presence of 1 mM FBP, 0.25 mM AMP, and 10 μM PLP. A control with no protein was also performed for all the samples. A continuous temperature increase from 20.0 to 99.0°C was scanned every 0.4°C in a ramp increment of 1.5°C per minute. Scans were run in triplicates and averaged. The unfolding temperatures (T_m_) of the proteins were measured using the minimum of the third derivative of the scanned fluorescence vs. temperature (d^3^F/dT^3^). Smoothing and differentiation of the thermograms were performed using a twenty-five-point Savitzky–Golay algorithm to determine the T_m_ (Savitzky and Golay, 1964). Traditionally, T_m_ has been determined by the minimum of the negative of the first derivative (–dF/dT). However, we preferred the use of higher derivatives because it is well-documented for spectrometry that it removes drifts, interferences, and enhances sharper peaks (Butler, 1979). Calculated T_m_ did not vary, but the graphical comparison of different thermal melting curves was clearer using d^3^F/dT^3^.

### Sequence Alignment

Amino acid alignment was performed with the program BioEdit 7.0.5.3 using the ClustalW algorithm incorporated into the software (Thompson et al., 1994; Hall, 1999).

### Ligand Modeling

Ligands were modeled into the tetramer (subunits I, J, K, L) of the *E. coli* ADP-Glc PPase (PDB ID: 5L6S). Subunits L and I were specifically used to model the ligands because they had FBP in the crystal structure. Ligands were docked into the putative regulatory sites in two stages. The first was a semi-manual placement of the molecule with respect to geometry, and the second was energy minimization. Stage I—It was assumed that the phosphate groups of the ligands were going into (1) the sulfate site that interacts with Arg52 and (2) the phosphate site from FBP that interacts with Lys39. The coordinates of these groups were copied and the coordinates of the rest of the atoms from the ligands FBP and HBP were optimized for angles and bond lengths. In the case of the ligand PLP covalently bound to Lys39, (2S)-2-amino-6-[[3-hydroxy-2-methyl-5-phosphonooxymethyl)pyridin-4-yl]methylideneamino]hexanoic acid (PDB code: LLP), the starting structure of this Schiff base compound was obtained from the PDB bank. LLP replaced Lys39 by copying the coordinates of the backbone and the β-carbon. The 5′-phosphate group was docked into the two putative phosphate-binding sites by copying the coordinates as described above. The rest of the atoms were placed maximizing optimal bond lengths and angles observed in the crystal structure. For all the ligands (FBP, HBP, and LLP), this initial optimization calculation was performed with Gnumeric 1.12 using the non-linear regression “Solver” utility. Stage II—After the first stage, only minor steric hindrances were observed apart from Arg130 in subunit I (which intrudes to the site from the neighboring subunit K). In subunit L, no such hindrance was observed from subunit J (Arg130 had a different rotamer). To avoid this issue, a different Arg130 rotamer was selected from the Dunbrack database (Shapovalov and Dunbrack, 2011) using the program Chimera 1.13.1 (Pettersen et al., 2004). This rotamer was chosen because it interacts with the phosphate neighboring Lys39. After this step, the structure was minimized with Chimera (Pettersen et al., 2004) with 100 steepest descent and 100 conjugate gradient steps (Wang et al., 2004). Minimization routines were from MMTK included with Chimera. Amber parameters were used for standard residues, and Amber's Antechamber module (Chimera) were used to assign parameters to non-standard residues (Wang et al., 2006).

### Molecular Dynamics Simulations

Starting with the same tetramer used for the ligand modeling described above, an independent modeling of the interactions between HBP and the protein was done using a combination of energy minimization and molecular dynamics. Each simulation box, containing one tetramer, a TIP3 water box extending at least 10 Å beyond the protein in all directions and 0.15 M NaCl adjusted to neutralize the charge in the water box, was assembled using the molecular graphics program VMD (Humphrey et al., 1996). The simulation box was then brought to equilibrium using the molecular dynamics program NAMD (Phillips et al., 2005). The equilibration procedure involved energy minimization with and without restraints on the protein coordinates (3,000 steps each), slow heating from 10 to 310 K (30,000 steps), and then pressure and temperature equilibration using a Langevin piston (10,000 steps) followed by unrestrained dynamics for 100,000 steps. Finally, the system was subjected to energy minimization for 3,000 steps to remove dynamically stretched bonds or angles. The time step was 2 fs with every 150th step being saved in the trajectory for analysis. Periodic boundary conditions were used. The cutoffs for non-bonding (van der Waals and electrostatic) interactions were 15 Å. The switch distance was 13 Å, and 1.0 1 ± 4 scaling factor was used. All calculations were done using CHARMM 36 parameters (Huang et al., 2017). Topology and parameter files for HBP were generated using the CHARMM General Force Field (CGenFF) (Vanommeslaeghe et al., 2012). The molecular graphics diagram was generated using VMD (Humphrey et al., 1996).

## Results

### Competition Between FBP and Sulfate

**Figure 2:**
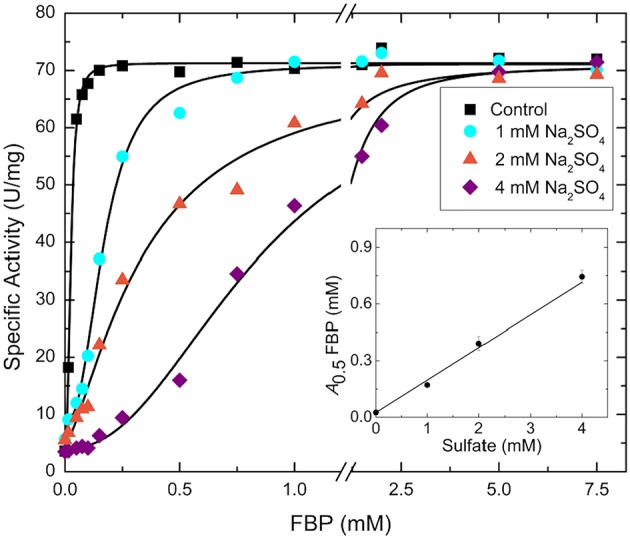
FBP saturation curves for the WT *E. coli* ADP-Glc PPase in the presence of varying concentrations of sulfate. Activity was measured in the presence of 1.0 mM Glc1P, 1.0 mM ATP, 7 mM MgCl_2_, and 50 mM HEPES pH 7.5. The effector and sodium sulfate concentration were varied as indicated in the figure. The inset graph represents the linear trend between the calculated *A*_0.5_ of FBP from each curve and the concentration of sulfate.

Sulfate and phosphate are isosteric, and the former could easily bind where the phosphoryl moiety of sugar phosphates bind with a similar type of interaction. We hypothesized that the FBP binding site involved in the activation of *E. coli* ADP-Glc PPase is partially located where a sulfate has been detected in the crystal structure (Cifuente et al., 2016). The FBP may have been displaced by the presence of sulfate (200 mM) in the crystallization conditions. To test if sulfate could have outcompeted FBP from the putative activator site, we performed kinetic assays to analyze the interaction between them (Figure 2). By varying concentrations of sodium sulfate and FBP, the activity of the enzyme revealed a typical competitive pattern. Relatively low concentrations of sulfate (4 mM) dramatically inhibited the enzyme when the concentration of FBP was just above the *A*_0.5_. However, in all cases, further increases of FBP outcompeted sulfate to reach similar *V*_max_ near 70 U/mg. In addition, as in a typical competitive process, the *A*_0.5_ for FBP varied linearly with sulfate concentration (Figure 2).

### Structural Feasibility of Activators to Bind Into Positively Charged Pockets

Considering that the activator FBP may bind where sulfate binds, we evaluated the structural feasibility of this hypothesis through modeling. Using the available crystal structure (Cifuente et al., 2016), we modeled FBP and structural analogs into a putative allosteric site that assumes a partial overlap with known sulfate and phosphate sites. An important aspect to consider is that FBP could exist in more than one configuration. Previous results strongly suggested that the linear rather than the cyclic furanose configuration was the activating form of FBP (Haugen and Preiss, 1979). It is known that 1,6-hexanediol bisphosphate (HBP), which is an analog of the linear form of FBP, is a stronger activator of the *E. coli* ADP-Glc PPase. The reported *A*_0.5_ was 4 μM with a higher maximal specific activity than the one observed with saturating FBP (Haugen and Preiss, 1979).

**Figure 3:**
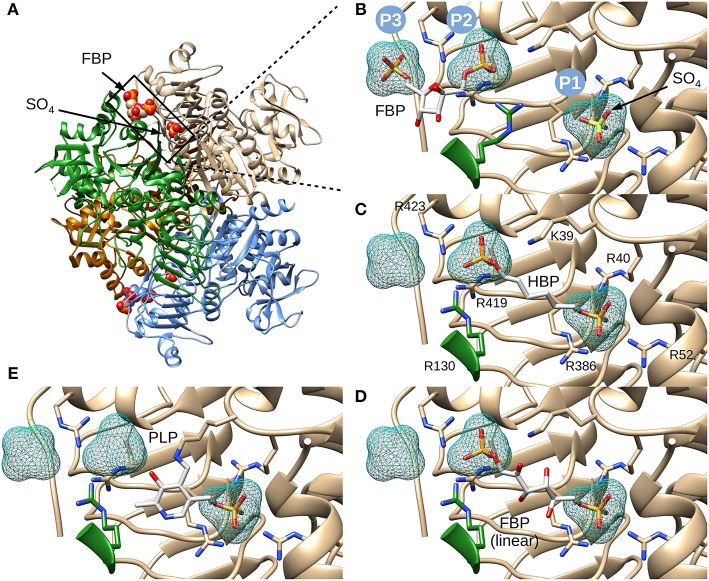
Structure of *E. coli* ADP-Glc PPase and positioning of various ligands. **(A)** Ribbon representation of homotetrameric *E. coli* ADP-Glc PPase in the presence of FBP and sulfate (PDB ID: 5L6S). **(B)** Localization of the FBP-binding site. P1 shows the accessible surface area of the sulfate in 5L6S. P2 and P3 designate the accessible surface area of modeled phosphates equivalent to the phosphate groups of FBP from 5L6S. **(C)** HBP modeled into P1 and P2. **(D)** The linear form of FBP modeled into the putative FBP binding site comprising P1 and P2. The 1- and 6-phosphate groups were modeled into P2 and P1, respectively. **(E)** PLP modeled into the putative regulatory binding site, forming a Schiff base with Lys39 and the phosphoryl moiety at P1.

We chose to model HBP in the putative allosteric site of the enzyme, not only because it is a known potent activator, but also because the molecule is linear and symmetrical (i.e., phosphate groups 1 and 6 are indistinguishable). This symmetry narrows the possibilities of how the molecule can be placed. While modeling this ligand, we hypothesized that the phosphate groups will fit well in places where sulfates or other phosphate groups have been found in the structure (Cifuente et al., 2016). Three positively charged pockets became suitable candidates to accommodate phosphoryl groups in the cleft between the N- and C-terminal domains (Figure 3A). One is where a sulfate is bound (P1), and the other two are where phosphates groups from FBP are located (P2 and P3) (Figure 3B). We were able to model HBP into the structure of *E. coli* ADP-Glc PPase restraining the phosphate groups to binding centers P1 and P2 (Figure 3C). The length of this molecule correctly fits between these pockets. These results were also confirmed through molecular dynamic simulation (Figure S1). The simulation showed that binding HBP between P1 and P2 would recruit more positively charged residues, particularly arginines, to the ligand than binding it between P2 and P3. At the end of the simulation, the Van der Waals and electrostatic interaction energy between the HBP and the protein, which was almost entirely electrostatic in this case, indicated much stronger binding at the P1-P2 site (−982 kcal/mole) than at the P2-P3 site (−550 kcal/mole).

The initial model with HBP also indicated that FBP could fit in the linear form between P1 and P2. This was certainly the case, and the presence of extra hydroxyl groups, in linear FBP, did not create steric hindrances (Figure 3D). This model docked the 6-phosphate group in the P1 site and the 1-phosphate group in the P2 site. However, orienting the FBP in the opposite direction also fit without steric hindrances (not shown). Slight movements of the Arg residues can also allow the binding of the cyclic form of FBP (not shown). Thus, we cannot discard this possibility.

PLP, which forms Schiff bases with lysine residues, is a powerful activator analog in various ADP-Glc PPases (Ballicora et al., 2003, 2004; Asencion Diez et al., 2014). In the *E. coli* enzyme, PLP forms a Schiff base with Lys39 (Parsons and Preiss, 1978a,b). Therefore, we modeled PLP in the allosteric site covalently bonded with Lys39 and with the phosphate placed in the P1 pocket (Figure 3E). All these models, including the activator FBP and analogs HBP and PLP, demonstrate that it is structurally and geometrically feasible for them to bind in the putative binding center P1.

### Scanning Mutagenesis

One common characteristic of these putative phosphate-binding centers (P1, P2, and P3) is that they are surrounded by arginines. Arginine residues are commonly found in the active sites of enzymes that bind phosphorylated compounds (O'Brien et al., 2008). It has been established that Arg can have strong electrostatic interactions with phosphates (Woods and Ferre, 2005), forming different types of Arg–phosphate complexes. They could form mono dentate and bidentate interactions (Yusufaly et al., 2014), “forks,” (Calnan et al., 1991) “claws,” (Hamelberg et al., 2007), or “clamps” (Komeda et al., 2011). As an allosteric site example, an Arg cluster has been recognized to be essential in binding the activator glucose 6-phosphate from a *Saccharomyces cerevisiae* glycogen synthase (Baskaran et al., 2010). Because of their potential significant role in phosphate group binding, we mutated all the Arg residues that surround centers P1, P2, and P3. In addition, we mutated Arg353 as a control. Though found in the interface between the C- and the N-terminal domain, Arg353 does not interact with any of these putative centers. Other residues mutated were: Arg40, Arg52, Arg386 (P1 center), Arg130, Arg419 (P2 center), and Arg423 (P2 and P3 center). After mutagenesis, we evaluated the ability of the enzyme to bind FBP and be activated.

### Effect of Mutations on Enzyme Activity

#### FBP Effects on ATP Apparent Affinity

**Figure 4:**
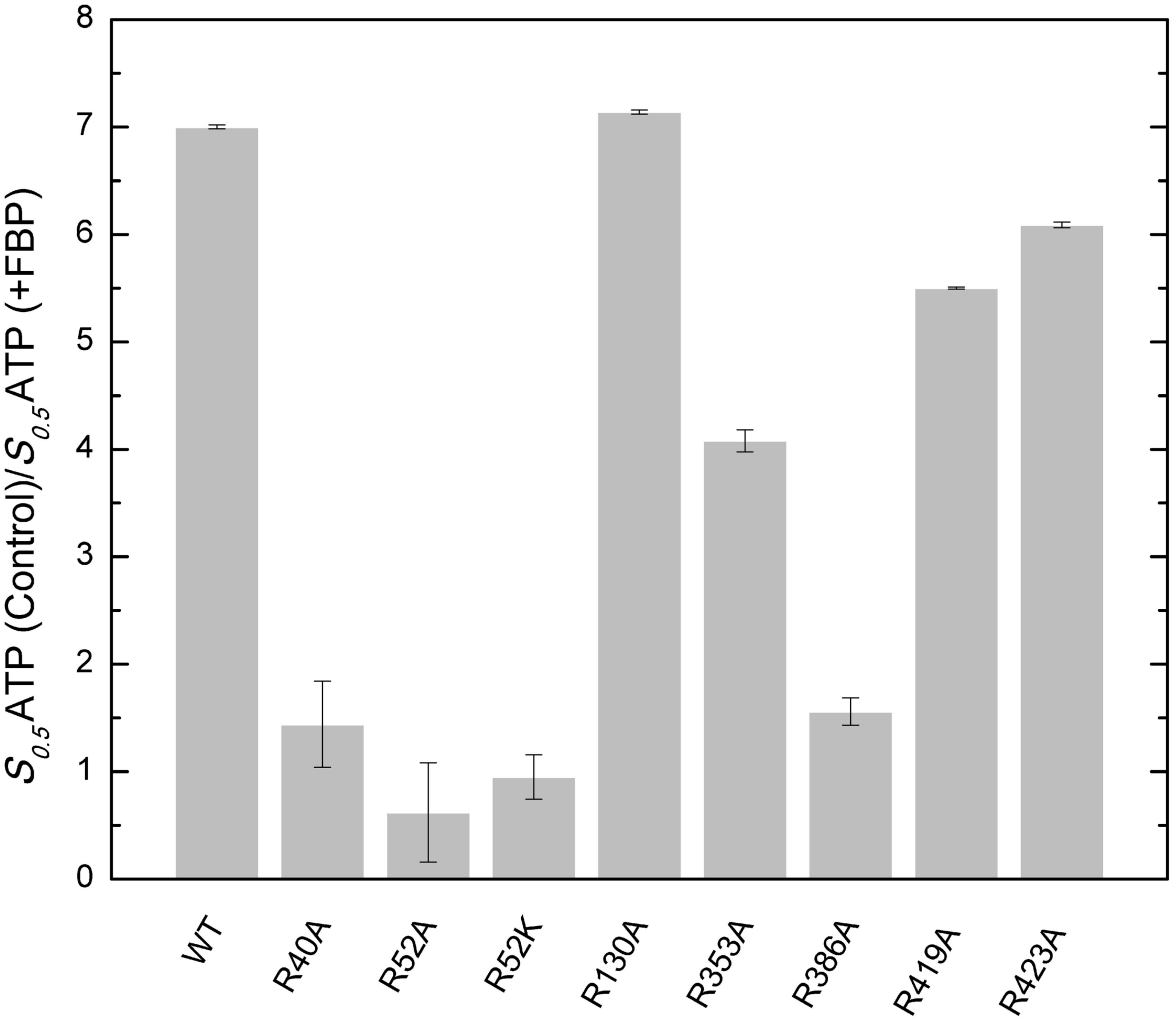
Effect of FBP on the apparent affinity for ATP in the WT and mutants of the *E. coli* ADP-Glc PPase. The effect of the FBP on the ATP apparent affinity was assessed by the ratio of two different *S*_0.5_ for ATP. One was measured in absence (control) and the other in presence of 1 mM FBP. Assays were performed in the presence of 1 mM Glc1P, 10 mM MgCl_2_, and 50 mM HEPES pH 8.0 as indicated in section Materials and Methods.

FBP significantly controls the binding of ATP in *E. coli* ADP-Glc PPase (Hill et al., 2015). Mutants R353A, R419A, and R423A had similar kinetic profiles to the WT (Table S1). FBP increased the apparent affinity for ATP at least 4-fold for these proteins (Figure 4). In addition, the presence of 1 mM FBP increased the specific activity at saturating concentrations of ATP more than 2-fold. On the other hand, R40A, R52A, R52K, and R386A exhibited a diminished response to the addition of FBP. Interestingly, R130A was pre-activated, with a basal activity (*v*_0_) of 54.0 U/mg in absence of activator (Table S1). This agrees with previously published data (Comino et al., 2017) but in addition, we found that the presence of saturating FBP increased the apparent affinity for ATP nearly 7-fold and the *V*_max_ to 71.5 U/mg.

#### FBP Effects on Specific Activity

**Figure 5:**
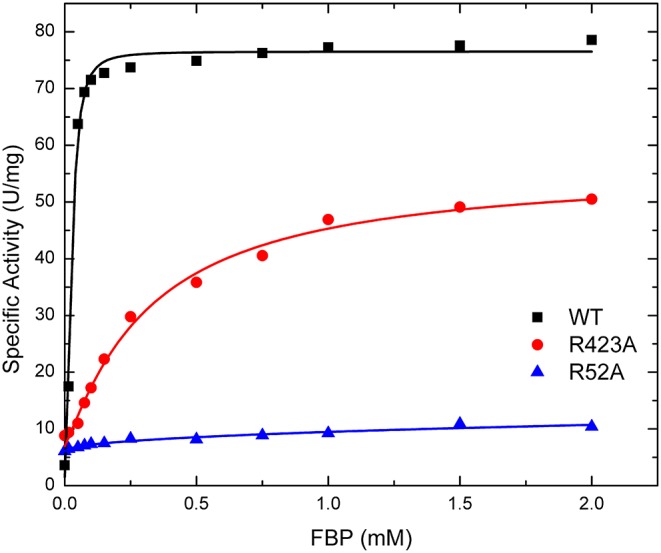
Effect of FBP on WT, R423A, and R52A *E. coli* ADP-Glc PPase activity. Activity was measured as described in section Materials and Methods in the presence of 1.0 mM Glc1P, 1.5 mM ATP, 10 mM MgCl_2_, and 50 mM HEPES pH 8.0. The effector concentration was varied as indicated in the figure.

The effect of FBP on the activity of the enzyme is mostly altered by the mutation of residues at the P1 site (Table 1). The activation of mutants R40A, R52A, and R52K are negligible as the concentration of FBP increases, while the WT and the other mutants exhibit a >6-fold response to the activator (*V*_max_*/v*_0_*)*. Mutant R423A had a weaker apparent affinity for FBP than the WT, but it was still able to activate the enzyme to reach a specific activity of 56.0 U/mg (Figure 5). On the other hand, R52A showed negligible activation by FBP, even at conditions that are saturating for WT.

**Table 1:** FBP activation parameters of *E. coli* ADP-Glc PPase wild-type and mutants.

**Enzyme^**a**^**	**FBP**				
	***A*_**0.5**_**	***n*_**H**_**	$v_{0}^{b}$	***V*_**max**_**	***V*_**max**_/*v*_**0**_**
	**(mM)**		**(U/mg)**	**(U/mg)**	**(-fold)**
WT	0.027 ± 0.009	1.34 ± 0.17	3.3 ± 1.7	76.0 ± 0.2	23.2
R40A	N/A^c^	N/A^c^	1.25 ± 0.02	1.41 ± 0.01	1.1
R52A	N/A^c^	N/A^c^	1.76 ± 0.10	2.14 ± 0.08	1.2
R52K	N/A^c^	N/A^c^	1.70 ± 0.09	2.16 ± 0.06	1.3
R130A	0.067 ± 0.010	1.33 ± 0.24	63.6 ± 2.1	87.7 ± 0.6	1.4
R353A	0.040 ± 0.010	0.97 ± 0.26	5.6 ± 1.8	54.6 ± 2.9	9.8
R386A	1.00 ± 0.13	1.08 ± 0.09	2.84 ± 0.18	18.5 ± 1.0	6.5
R419A	0.036 ± 0.011	1.28 ± 0.4	11.0 ± 4.0	66.4 ± 3.1	6.0
R423A	0.32 ± 0.05	1.12 ± 0.19	7.0 ± 2.1	56.0 ± 3.8	8.0

a Assays were performed as described in section Materials and Methods.

^b^v_0_ is the activity in absence of activator.

c *Activation was not significant enough to determine the parameter with precision or justify significance*.

#### PLP Activation

**Figure 6:**
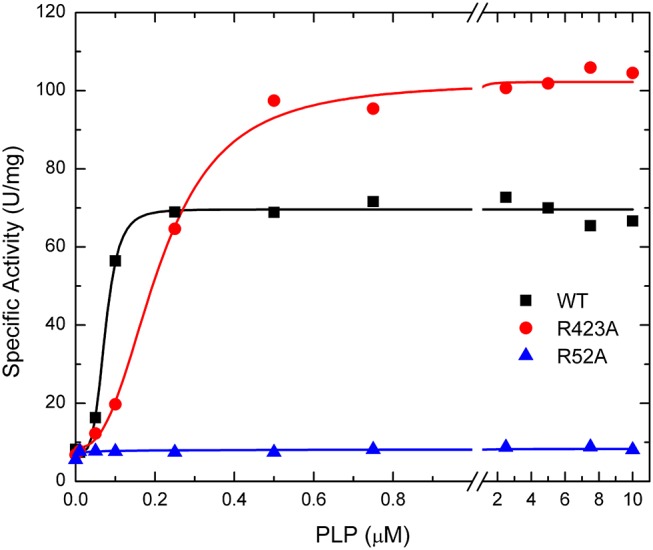
Effect of PLP on WT, R423A, and R52A *E. coli* ADP-Glc PPase activity. Activity was measured as described in Materials and Methods in the presence of 1.0 mM Glc1P, 1.5 mM ATP, 10 mM MgCl_2_, and 50 mM HEPES pH 8.0. The effector concentration was varied as indicated in the figure.

The activation effect of PLP was disturbed by mutating residues at the P1 site (Table 2). PLP activated the WT extremely well, with an *A*_0.5_ of 0.075 μM, an 8.6-fold increase in activity at saturating concentrations, and a high degree of cooperativity (*n*_H_ ~ 4, Table 2). On the other hand, PLP activated mutants R40A, R52A, R52K, and R130A <2.3-fold, whereas it increased the activity in the other mutants at least 4.5-fold at saturating concentrations. Interestingly, PLP increased the activity of R423A 13.5-fold, but its apparent affinity was lower than the wild type enzyme (Figure 6).

**Table 2:** PLP activation parameters of *E. coli* ADP-Glc PPase wild type and mutants.

**Enzyme^**a**^**	**PLP**				
	***A*_**0.5**_**	***n*_**H**_**	$v_{0}^{b}$	***V*_**max**_**	***V*_**max**_/*v*_**0**_**
	**(μM)**		**(U/mg)**	**(U/mg)**	**(-fold)**
WT	0.075 ± 0.004	4.55 ± 0.67	8.1 ± 2.3	69.7 ± 0.2	8.6
R40A	0.48 ± 0.22	0.67 ± 0.29	6.8 ± 0.5	10.2 ± 0.6	1.5
R52A	N/A^c^	N/A^c^	7.2 ± 3.3	8.6 ± 2.8	1.2
R52K	0.22 ± 0.05	1.27 ± 0.34	4.1 ± 0.5	9.6 ± 0.3	2.3
R130A	1.29 ± 0.23	1.83 ± 0.50	56.5 ± 1.1	80.8 ± 1.8	1.4
R353A	0.16 ± 0.01	3.3 ± 0.5	10.9 ± 1.2	87.2 ± 1.3	9.3
R386A	4.2 ± 0.6	3.0 ± 0.9	15.3 ± 1.1	68.2 ± 6.3	4.5
R419A	0.019 ± 0.005	3.2 ± 1.0	11.2 ± 1.2	51.8 ± 0.9	4.6
R423A	0.21 ± 0.01	2.61 ± 0.34	8.7 ± 2.5	102.0 ± 1.4	13.5

a Assays were performed as described in section Materials and Methods.

^b^v_0_ is the activity in absence of activator.

c *Activation was not significant enough to determine the parameter with precision or justify significance*.

#### AMP Inhibition

Prior studies established that FBP competes with AMP in the *E. coli* ADP-Glc PPase (Gentner and Preiss, 1968). The major inhibitory effect of AMP is to compete with FBP preventing its activation. In absence of FBP, the enzyme is relatively insensitive to AMP inhibition (Gentner and Preiss, 1968; Figueroa et al., 2011). In addition, HBP competes with the binding of both AMP and FBP, indicating that all three effectors have overlapping sites (Haugen and Preiss, 1979). Therefore, we analyzed the effect of the mutations on the inhibition kinetics. In this respect, the effects correlated and were in good agreement with the previous results from the Preiss group. The mutants that lack response to FBP were also resistant to AMP inhibition by retaining at least 66% of their original activity. At saturating levels of AMP, the WT, R353A, R419A, and R423A only had <5% of its original activity (not shown).

### Effect of Mutations on Ligand Binding

#### FBP Binding

**Figure 7:**
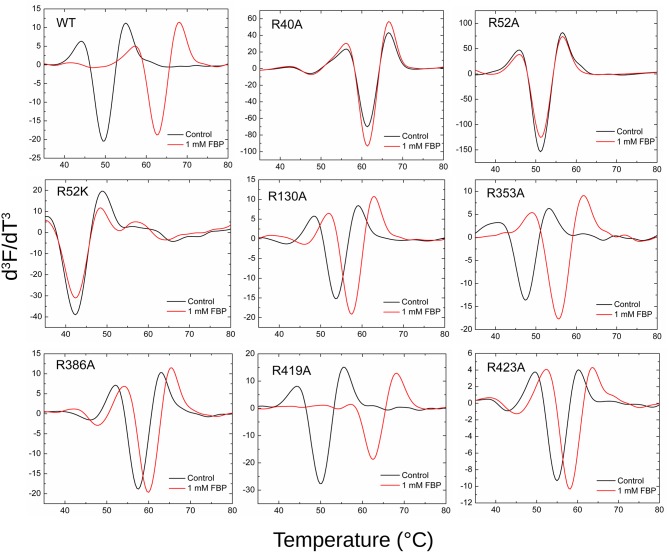
Effect of FBP on the thermal shift assays for the WT and mutants of ADP-Glc PPase. Thermal shift assays were performed as described in Materials and Methods in presence and absence (control) of FBP as indicated.

Thermal shift assays, which indicate the ability of a ligand to bind to the protein, supported the kinetic results (Figure 7). The presence of 1 mM FBP shifted the WT T_m_ 13.2°C, whereas it had no apparent effect on R40A, R52A, and R52K (Table S2). On the other hand, FBP shifted the T_m_ of R353A and R419A by 8.1 and 12.2°C, respectively. The other mutants (R130A, R386A, and R423A) had intermediate but significant thermal shifts >2.4°C.

#### PLP Binding

The mutants that are activated by PLP (R353A, R419A, R423A, R130A) also showed thermal shifts in the presence of 10 μM PLP, whereas R40A, R52A, R52K, and R386A had <1°C shift (Table S2, Figure S2).

#### AMP Binding

Thermal shift assays confirmed the kinetic results showing no apparent binding of 1 mM AMP to R40A, R52A, R52K, and R386A, whereas the WT and mutants that responded to AMP inhibition exhibited clear thermal shifts >8°C (Table S2, Figure S3).

## Discussion

The location of the activator sites in ADP-Glc PPases is the structural basis to understand how the synthesis of bacterial glycogen and starch in plants is allosterically regulated. Recently, structures with FBP (*E. coli*) and pyruvate (*A. tumefaciens*) have been reported (Cifuente et al., 2016; Hill et al., 2019). Due to the positioning of FBP in the published structure of *E. coli* ADP-Glc PPase, we further analyzed the impact of sulfate on FBP activation. Kinetic analysis of the enzyme, in presence of both sulfate and FBP, showed that sulfate behaves like a competitor of FBP (Figure 2). We postulate that the binding location of FBP, as reported (Cifuente et al., 2016), may be an alternative binding site when there are competing sulfate molecules in solution. Sulfate will bind at high concentrations to pocket P1, forcing FBP to relocate its phosphate groups to pockets P2 and P3 (Figure 3). The affinity for sulfate to bind to pockets P2 and P3 may be much lower than P1. In the unit cell, no sulfate molecules were detected in pockets P2 and P3 from the subunits that did not bind FBP (Cifuente et al., 2016).

By mapping the potential residues involved in FBP binding, we can further understand the allosteric regulation of ADP-Glc PPase. We examined the FBP binding site through a mutagenesis scanning of arginines in the C- and N-terminal domains of ADP-Glc PPase. We made alanine mutants of the following residues: Arg40, Arg52, Arg130, Arg353, Arg386, Arg419, and Arg423. Of those, R40A, R52A, and R386A significantly disrupt the allosteric response. R40A and R52A have negligible activation by either FBP or PLP, indicating that Arg40 and Arg52 are essential for regulation. R386A is still activated by FBP and PLP, but their apparent affinities are 37- and 53-fold lower, respectively (Tables 1, 2). Arg52, which seems to be one of the most critical residues studied, was further characterized with a mutation to lysine to probe whether a positive charge can restore function. Despite this replacement, R52K has a very similar kinetic profile to the R52A enzyme. It has no activation response and near negligible binding toward FBP, and very slight activation by PLP. The fact that Lys does not restore the functionality of Arg in this position suggests that the geometry given by the side chain of Arg is important, not only the charge (Schug and Lindner, 2005). Thermal shift assays confirmed that R423A, R419A, and to a lesser extent R386A, were stabilized by FBP. However, mutants of Arg40 and Arg52 did not have a significant thermal shift with FBP meaning these residues are critical for FBP binding (Figure 7). Interestingly, the replacement of Arg side chains involved in the activation with Ala yielded mutants more stable than the WT, even in absence of FBP (Figure 7). The T_m_ of R40A, R386A, R423A, R130A, R52A were higher than the WT by 11.8, 7.9, 5.4, 4.1, 1.7°C, respectively (Figure 7). Residues Arg353 and Arg419 do not participate in the activation and their Ala mutants were not significantly more stable than the WT. All these results suggest that an accumulation of positive charges in the activator pocket de-stabilizes the enzyme. Removal of the positive charge either by mutagenesis or binding to a negatively charged activator stabilizes the enzyme.

The recent crystal structure of the *E. coli* ADP-Glc PPase showed R419 and R423 interacting with FBP (Cifuente et al., 2016). However, the bound FBP had no apparent interactions with Arg40, Arg52, and Arg386. The mutagenesis and binding data point to the importance of these three arginine residues. Thus, we postulate that FBP activates by going into pockets P1 and P2. Residues Arg40, Arg52, and Arg386 (P1 site) would cradle one phosphate group of FBP, whereas Arg423 and Lys39 (P2 site) would accommodate the other one. Though we cannot claim which of the phosphate groups from FBP would go to which site (P1 or P2), structurally, both options seem feasible. Because of the symmetry, this is not an issue for HBP, which is a known activator analog (Haugen and Preiss, 1979). It has been previously proposed that the activating form of FBP could be linear (Haugen and Preiss, 1979). Our results fit this scenario, but we cannot discard the possibility that the cyclic form activates the enzyme.

PLP has two major types of interaction with proteins. One is through the aldehyde, which makes Schiff bases with Lys, and the other is through the 5′-phosphate group that makes strong salt bridges. It is known that PLP forms a Schiff base with Lys39 in the *E. coli* ADP-Glc PPase (Parsons and Preiss, 1978a,b). This was critical information when modeling PLP binding because it limited the number of alternative conformations. We explored the possible binding scenarios for PLP, assuming a Schiff base formed with Lys39. There were no obvious constraints for the phosphate to go to either the P1 or the P2 site (Figure S4). Both these conformations could be accommodated with no steric hindrances. However, considering Arg52 is essential for PLP activation and Arg423 is not (Figure 6), we interpret that PLP activates when the phosphate is anchored at the P1 site (Figure S4). Interestingly, the maximum activity with PLP of the mutant R423A is higher than the wild type (Figure 6). This could be explained if the phosphate of PLP swings between both the P1 and P2 sites. If in the wild type enzyme, a fraction of PLP is interacting with Arg423 (P2, unproductive binding, inactive) but the majority with Arg52 (P1, productive binding, active), the total effect will still be activation, but suboptimal. R423A would weaken the binding to the P2 site, shifting the phosphate-binding equilibrium toward Arg52 (P1). Therefore, PLP can bind more productively in the mutant R423A. Consequently, this mutant becomes a more active form than the wild type in the presence of PLP.

We have previously shown that certain mutations in the loop Pro103-Arg115 of the *E. coli* ADP-Glc PPase disrupt the allosteric response but not the binding of the activators (Hill et al., 2015). This is because they are responsible for triggering the allosteric signal. Nevertheless, this is not the case with the mutants studied here. Based on the kinetic and binding experiments, we can conclude that Arg40, Arg52, and Arg386 form a pocket that is responsible for the binding of a phosphate moiety of an activator (FBP, HBP, and PLP). In this study, because the binding of activators is severely affected in these mutants, no allosteric signal is triggered. For this reason, activation is not observed.

X-ray crystallography shows that AMP binds the *E. coli* ADP-Glc PPase by docking its phosphate group to the site that we here describe as P1 (Comino et al., 2017). Interestingly, the AMP structure was obtained in conditions with no sulfate in the medium that could potentially compete for the P1 site (Comino et al., 2017). Our results strongly suggest that FBP anchors one of its phosphate to the P1 pocket, explaining the competitive nature of their interaction. Thermal shifts assay confirmed previous results that Arg40 and Arg386 are essential for AMP binding, even more than Arg130 (Comino et al., 2017). Our results show that Arg52 is also critical for the binding of AMP (Figure S3). None of the other Arg mutants tested seems to have affected the binding (Figure S3).

The Arg residues investigated in this study are at the interface between the N- and C-terminal domains of one subunit and the N-terminal domain of a neighboring subunit (Figures 3A,B). The mutant of one of them, Arg130, displays a distinct behavior. R130A exhibits higher activity (63.6 U/mg) than the WT (3.3 U/mg) in absence of the activator (Table 1). With the addition of saturating concentrations of FBP, both the WT and R130A reach similar levels of activity (76.0 and 87.7 U/mg, respectively). This behavior could be interpreted based on the Monod-Wyman-Changeux (MWC) model of allosterism (Monod et al., 1965), whereby an inactive form (T) is in thermodynamic equilibrium with an active form (R), related by constant (L). In this model, the activator binds to the R form with higher affinity than to the T form, causing an increase in the population of R forms in solution. In the case of the *E. coli* ADP-Glc PPase, replacing Arg130 with Ala may make the R form more stable than T. This would cause an increase in the concentration of R, even in the absence of activator, explaining the pre-activated behavior of R130A. Under only this assumption, it would also be expected that the *A*_0.5_ of R130A decreases, because there is more of the R form for the activator to bind to with a high affinity. However, this was not observed. Most likely this is because Arg130 is predicted to interact with the phosphate in pocket P2, therefore, mutating this residue lowers the binding affinity. The modest increase in *A*_0.5_ observed (0.027 to 0.067 mM) in R130A is probably due to these competing effects. That is, R130A favors the R form, but at the same time, disturbs the interaction of the R form with the activator. The MWC model is also useful in explaining the cooperativity observed with the mutants. Whenever the *A*_0.5_ for FBP or PLP of one of the mutants increases, the *n*_H_ tends to be lower than the WT. Under this model, the observed cooperativity increases when “*c*,” the ratio between *K*_R_ and *K*_T_ (dissociation constants for the R and T form, respectively) increases. For these mutants, the *K*_R_ may have increased because the interaction between the activator and the R form is selectively altered causing “*c*” to increase, thereby decreasing *n*_H_. This is the trend observed with several of the mutants and their interaction with the activators FBP and PLP (Tables 1, 2).

**Figure 8:**
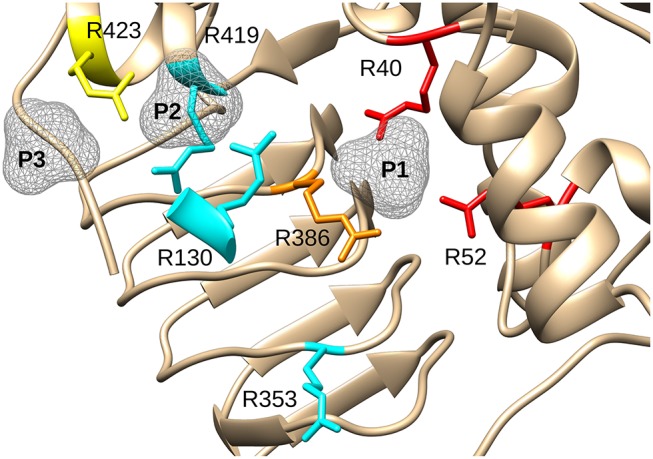
Effect of mutagenesis on the allosterism of the *E. coli* ADP-Glc PPase. Arg residues depicted in the figure were mutated to Ala and characterized as described in the text. In red (Arg40, Arg52), are residues with negligible activation after mutagenesis. In orange (Arg386), is the residue that displayed a significantly lower apparent affinity for FBP and PLP (37- and 56-fold, respectively). In yellow (Arg423), is the residue that displayed a near WT maximum activation but had 12-fold lower apparent affinity for FBP and higher activity with PLP (only 2.6-lower apparent affinity). In cyan, are Arg residues that did not display dramatic effects on the allosteric activation when they were mutated to Ala. P1, P2, and P3 are surfaces depicting putative binding sites for phosphate groups.

**Figure 9:**
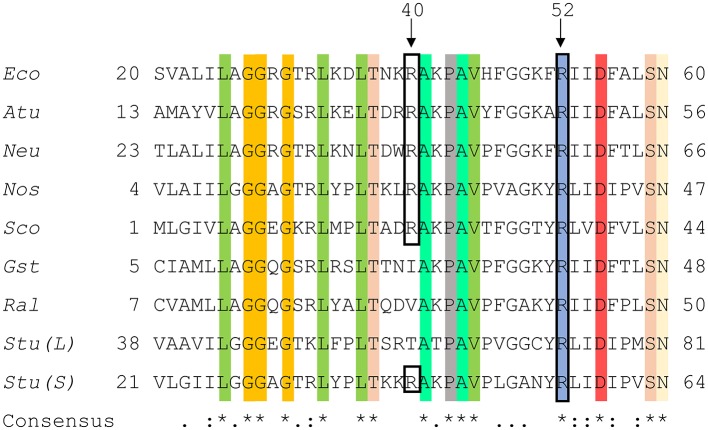
Sequence alignment of ADP-Glc PPases from different species. Amino acid alignment of ADP-Glc PPases from selected species were processed as described in section Materials and Methods. The accession numbers were attained from the NCBI database. Eco, *Escherichia coli* (P0A6V1.2); Atu, *Agrobacterium tumefaciens* (P39669.1); Neu, *Nitrosomonas europaea* (Q82T88.1); Nos, *Nostoc sp. PCC 7120* (P30521.1); Sco, *Streptoyces coelicolor* (CAA61885); Gst, *Geobacillus stearothermophilus* (O08326.1); Ral, *Ruminococcus albus* (WP_024858442.1); Stu (L), *Solanum tuberosum* (large subunit) (P55242.1); Stu (S), *Solanum tuberosum* (small subunit) (P23509.2). Highlighted regions display the conservation of amino acids amongst the selected organisms and the black box shows N-terminal residues involved in phosphate binding at the P1 site.

A structure with Arg residues that have been color-coded according to their activation contribution highlights an important region for allosterism (Figure 8). Arg residues that are more important face the N-terminal domain and the P1 site (Figure 8). This study provides experimental evidence that Arg40 and Arg52 in *E. coli* ADP-Glc PPase could potentially play a profound role in sugar phosphate activation. These residues are homologous to Arg33 and Arg45 in ADP-Glc PPase from *A. tumefaciens*, which were demonstrated to be important for Fru6P activation in that species (Gomez-Casati et al., 2001). More work in ADP-Glc PPases from other species will be needed to evaluate their broader importance. An alignment of ADP-Glc PPases from representative species shows Arg52 is conserved (Figure 9). On the other hand, Arg40 is missing in a few of them, which are enzyme forms not activated by a sugar phosphate. For instance, the enzymes from the Bacillus family are not allosterically regulated (Takata et al., 1997), the subunit from *Ruminococcus albus* is poorly activated by phosphoenolpyruvate (Cereijo et al., 2018), and the large (L) subunit from potato (*Solanum tuberosum*) tuber, has a defective regulatory site (Figueroa et al., 2011). The role of the L subunit in that particular enzyme is not to bind regulators *per se*, but rather to interact with the small (S) subunit, thereby altering its regulatory properties (Ballicora et al., 1998, 2005). Further studies on homologous enzymes from other species regulated by different sugar phosphates will provide more information about the structural and functional conservation of this regulatory site. This will be important to understand how the regulation has evolved to satisfy different metabolic needs, not only in bacteria, but also in plants.

## Data Availability Statement

The datasets generated for this study are available on request to the corresponding author.

## Author Contributions

MB designed the research and contributed new reagents or analytic tools. JB, BH, and AS performed the experiments. MB and KO performed computational research. JB, BH, AI, KO, and MB analyzed the data. JB, AI, and MB wrote the paper.

### Conflict of Interest

The authors declare that the research was conducted in the absence of any commercial or financial relationships that could be construed as a potential conflict of interest.

## Abbreviations

Glc1P: glucose 1-phosphate
ADP-Glc: ADP-glucose
FBP: fructose 1,6-bisphosphate
PLP: pyridoxal 5′-phosphate
HBP: 1,6-hexanediol bisphosphate
Fru6P: fructose 6-phosphate
PPi: pyrophosphate
LLP: (2S)-2-amino-6-[[3-hydroxy-2-methyl-5-(phosphonooxymethyl)pyridin-4-yl]methylideneamino]hexanoic acid.
