# Supplementary material for: Mapping of a Regulatory Site of the Escherichia coli ADP-Glucose Pyrophosphorylase
Source: Front Mol Biosci. 2019 Sep 25;6:89. doi: 10.3389/fmolb.2019.00089 (PMC6773804; doi:10.3389/fmolb.2019.00089)
Supplement: Supplementary file 1 [file Data_Sheet_1.PDF]

Table S1

Comparison of the V_{max} and ATP $S_{0.5}$ for wild type and mutant *E. coli* ADP-Glc PPases

Enzyme [a]	ATP (control)			ATP (+1 mM FBP)		
	$S_{0.5}$	n_H	V_{max}	$S_{0.5}$	n_H	V_{max}
	(mM)		(U/mg)	(mM)		(U/mg)
WT	1.75 ± 0.09	2.56 ± 0.28	24.6 ± 1.1	0.25 ± 0.02	1.93 ± 0.21	84.2 ± 2.1
R40A	3.5 ± 1.4	1.40 ± 0.24	6.9 ± 0.8	2.43 ± 0.43	1.63 ± 0.20	8.7 ± 2.3
R52A	1.38 ± 0.13	2.50 ± 0.50	4.99 ± 0.35	2.23 ± 0.43	1.35 ± 0.15	7.22 ± 0.81
R52K	2.58 ± 0.41	2.42 ± 0.57	11.8 ± 1.8	2.71 ± 0.13	3.01 ± 0.27	12.4 ± 0.7
R130A	1.00 ± 0.07	2.15 ± 0.29	54.0 ± 2.4	0.14 ± 0.01	1.36 ± 0.07	71.5 ± 0.8
R353A	0.98 ± 0.14	1.47 ± 0.21	27.7 ± 2.0	0.24 ± 0.10	0.64 ± 0.13	57.7 ± 3.6
R386A	1.56 ± 0.20	2.51 ± 0.64	3.40 ± 0.34	1.00 ± 0.07	2.41 ± 0.30	15.2 ± 0.6
R419A	1.97 ± 0.05	2.34 ± 0.77	25.2 ± 0.19	0.36 ± 0.01	2.73 ± 0.27	59.6 ± 1.0
R423A	1.40 ± 0.04	2.26 ± 0.33	23.8 ± 0.5	0.23 ± 0.03	1.70 ± 0.13	58.9 ± 2.6

[a] Assays were performed as described in Materials and Method

Table S2

Thermal shift assay of *E. coli* ADP-Glc PPase wild type and mutants with varying effectors

**Enzyme** [a]	**ΔT_m (˚C)**		
	**FBP**	**AMP**	**PLP**
	(1 mM)	(0.25 mM)	(10 µM)
WT	13.2 ± 0.1	11.9 ± 0.1	8.1 ± 0.2
R40A	0.00 ± 0.04	0.09 ± 0.06	0.23 ± 0.20
R52A	0.00 ± 0.08	0.03 ± 0.14	0.58 ± 0.17
R52K	0.40 ± 0.16	0.20 ± 0.12	0.3 ± 0.08
R130A	3.80 ± 0.07	1.7 ± 0.1	1.9 ± 0.2
R353A	8.10 ± 0.09	9.4 ± 0.08	5.7 ± 0.1
R386A	2.40 ± 0.07	0.08 ± 0.14	0.57 ± 0.15
R419A	12.2 ± 0.08	10.6 ± 0.2	7.3 ± 0.2
R423A	3.2 ± 0.08	8.9 ± 0.05	3.4 ± 0.04

[a] Assays were performed as described in Materials and Methods

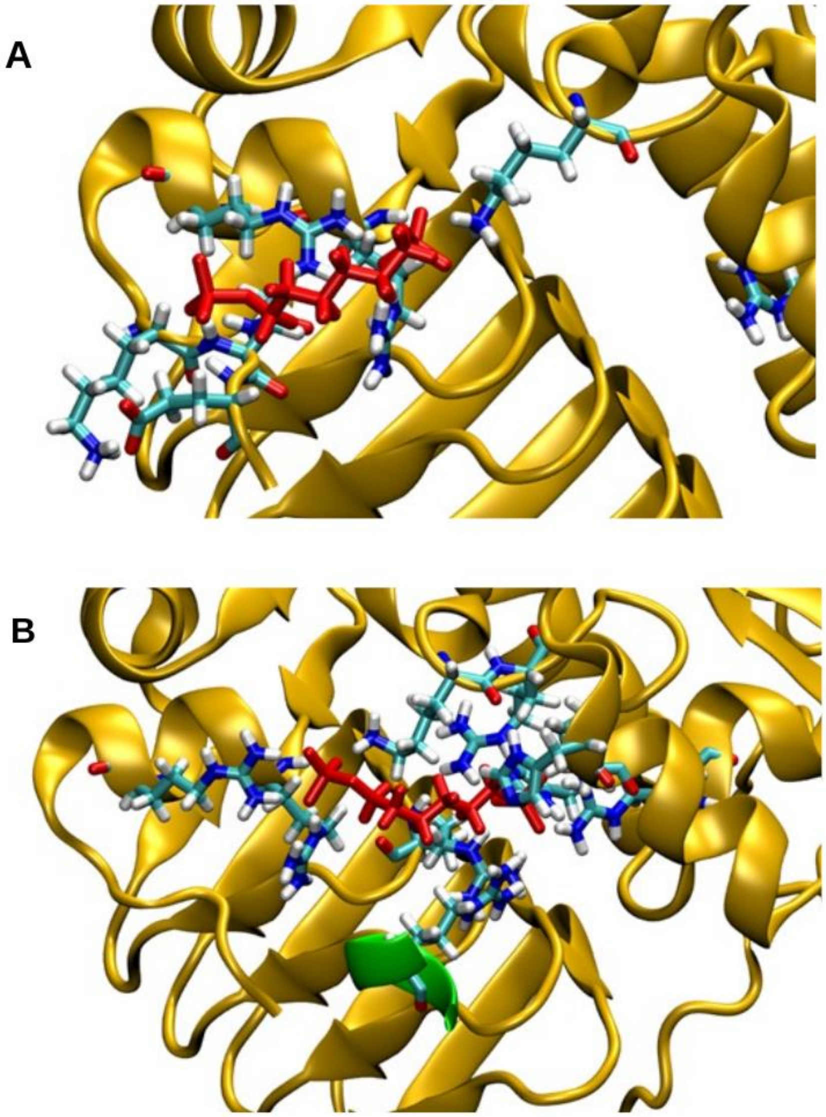

Fig. S1. Models of HBP binding sites generated by molecular dynamics simulations. HBP is shown in red. The residues shown all have at least one atom within 3 Å of the HBP. (A) This model was obtained by initially placing phosphate groups of the HBP in the P2 and P3 sites. In addition, Arg52 is shown at the right side of the figure to indicate where the P1 site is. (B) This model was obtained by initially placing phosphate groups of the HBP in the P1 and P2 sites. More residues that are positive were recruited to this HBP binding site than to the P2-P3 one, including one from the adjacent subunit shown in green.

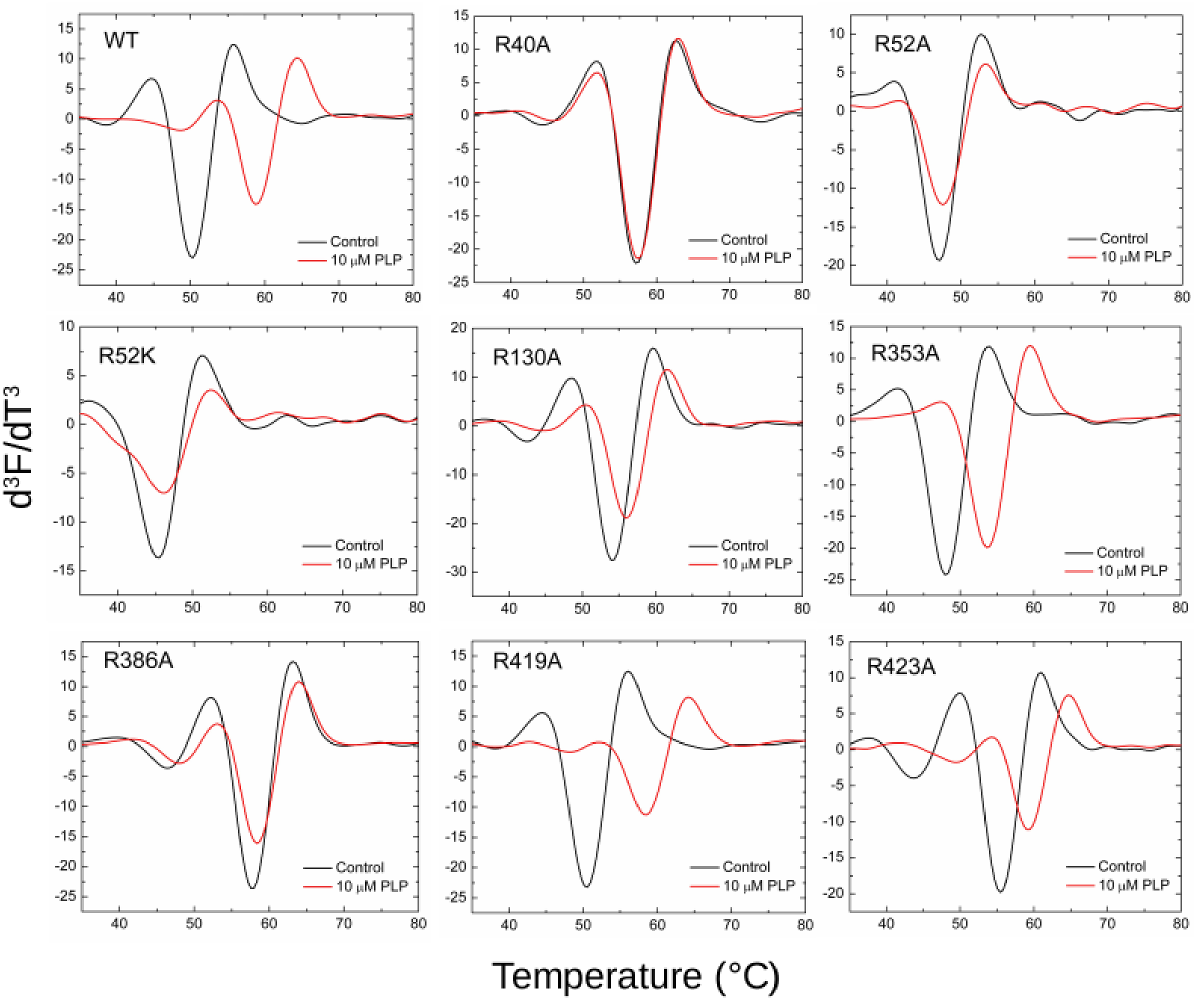

Fig. S2. PLP Thermal shift assays for the WT and mutants of *E. coli* ADP-Glc PPase. Thermal shift assays were performed as described in Materials and Methods.

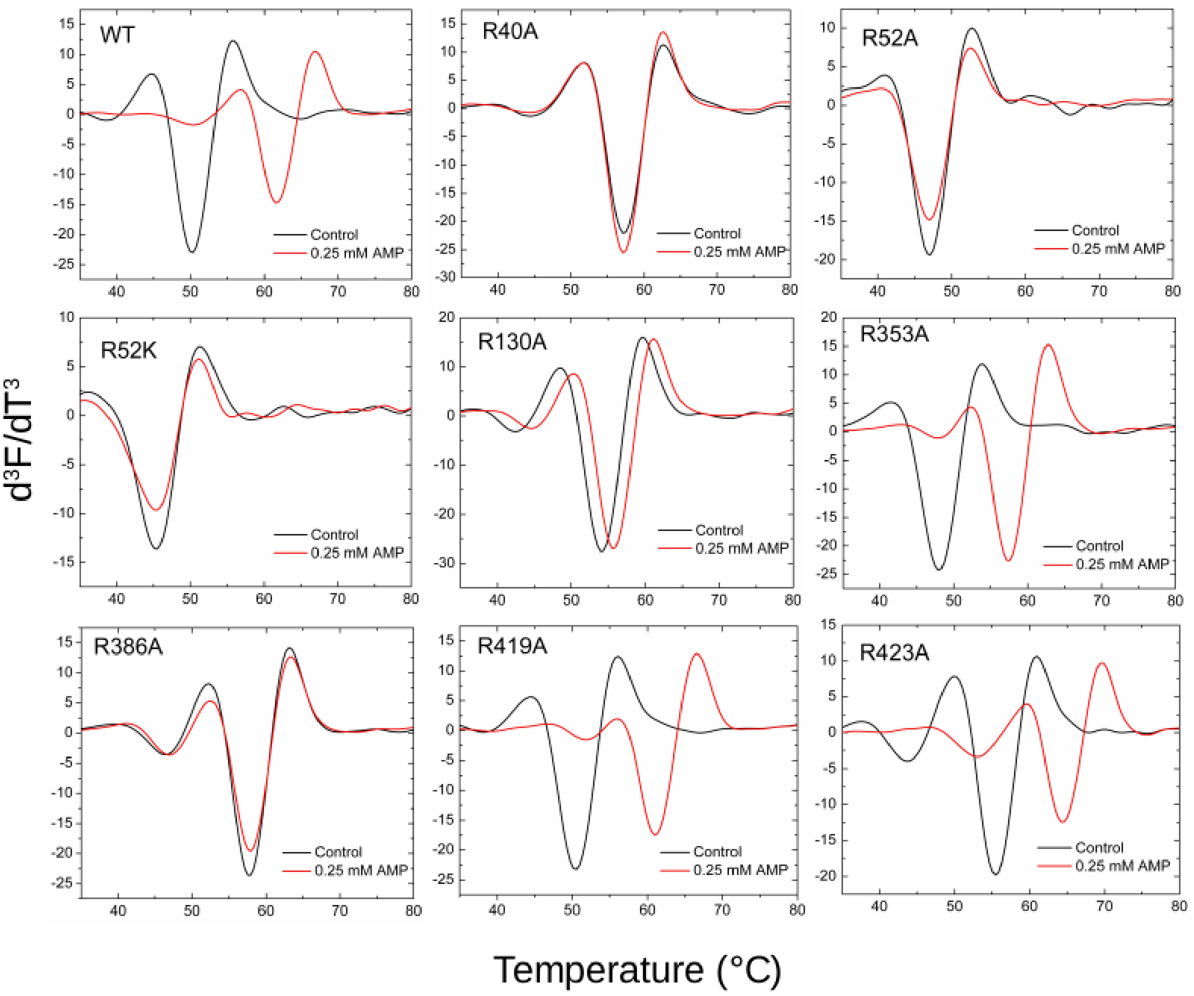

Fig. S3. AMP Thermal shift assays for the WT and mutants of *E. coli* ADP-Glc PPase. Thermal shift assays were performed as described in Materials and Methods.

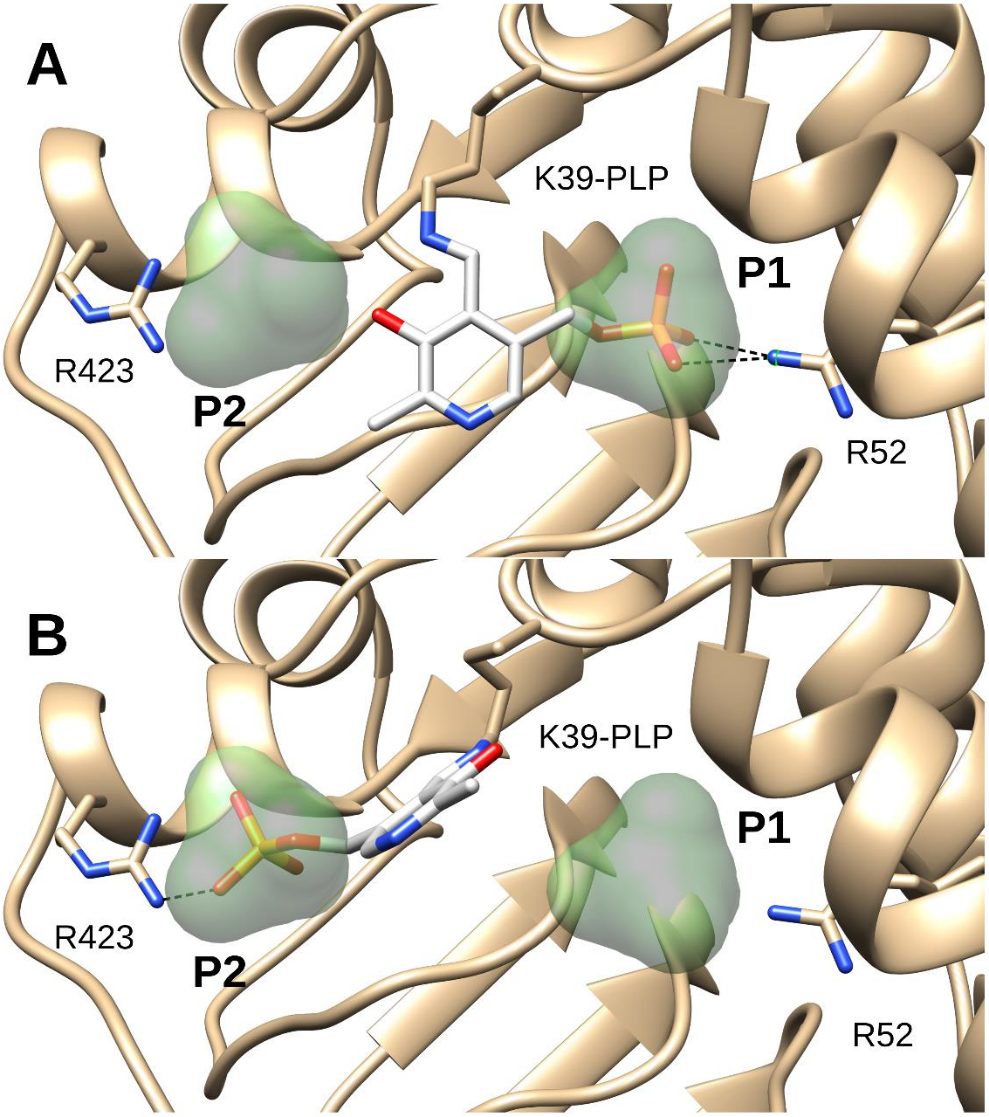

Fig. S4. Modeling of alternative binding modes of PLP to the *E. coli* ADP-Glc PPase. PLP was modeled assuming the formation of a Schiff base between the aldehyde of PLP and the ε-N of Lys39. In panel A, one alternative conformation placed the phosphate group in the P1 site interacting with Arg52. In panel B, the other conformation placed the phosphate in the P2 site interacting with Arg423.
